# LncRNA ZEB1‐AS1 down‐regulation suppresses the proliferation and invasion by inhibiting ZEB1 expression in oesophageal squamous cell carcinoma

**DOI:** 10.1111/jcmm.14692

**Published:** 2019-10-22

**Authors:** Yan Zhao, Ning Wang, Xiaosan Zhang, Hongtao Liu, Shujun Yang

**Affiliations:** ^1^ Department of Medical Oncology Affiliated Cancer Hospital of Zhengzhou University Zhengzhou China; ^2^ Henan Academy of Medical and Pharmaceutical Sciences Zhengzhou University Zhengzhou China; ^3^ College of Life Sciences Zhengzhou University Zhengzhou China

**Keywords:** Epithelial‐mesenchymal transition, long non‐coding RNA, oesophageal squamous cell carcinoma, ZEB1, ZEB1‐AS1

## Abstract

Multiple studies have unveiled that long non‐coding RNAs (lncRNAs) play a pivotal role in tumour progression and metastasis. However, the biological role of lncRNA ZEB1‐AS1 in oesophageal squamous cell carcinoma (ESCC) remains under investigation, and thus, the current study was to investigate the functions of ZEB1‐AS1 in proliferation and invasion of ESCC. Here, we discovered that ZEB1‐AS1 and ZEB1 were markedly up‐regulated in ESCC tissues and cells relative to their corresponding normal control. ZEB1‐AS1 and ZEB1 overexpressions were both related to TNM staging and lymph node metastasis as well as poor prognosis in ESCC. The hypomethylation of ZEB1‐AS1 promoter triggered ZEB1‐AS1 overexpression in ESCC tissues and cells. In addition, ZEB1‐AS1 knockdown mediated by siRNA markedly suppressed the proliferation and invasion in vitro in EC9706 and TE1 cells, which was similar with ZEB1 siRNA treatment, coupled with EMT alterations including the up‐regulation of E‐cadherin level as well as the down‐regulation of N‐cadherin and vimentin levels. Notably, ZEB1‐AS1 depletion dramatically down‐regulated ZEB1 expression in EC9706 and TE1 cells, and ZEB1 overexpression obviously reversed the inhibitory effects of proliferation and invasion triggered by ZEB1‐AS1 siRNA. ZEB1‐AS1 shRNA evidently inhibited tumour growth and weight, whereas ZEB1 elevation partly recovered the tumour growth in ESCC EC9706 and TE1 xenografted nude mice. In conclusion, ZEB1‐AS1 overexpression is tightly involved in the development and progression of ESCC, and it exerts the antitumour efficacy by regulating ZEB1 level in ESCC.

## 1 INTRODUCTION

Oesophageal cancer (ESCA) is considered as a high malignant neoplasm harbouring two main subtypes: oesophageal adenocarcinoma (EAC) and oesophageal squamous cell carcinoma (ESCC).^1^, ^2^ ESCC accounts for more than 90% in all ESCA, and its distribution mainly derives from eastern Asia, African, etc, especially in Lin County, Henan Province.^3^, ^4^, ^5^ Although tremendous advance in diagnosis and therapy, 5‐year survival rates of ESCA patients remain relative depressing, only exhibiting less than 20%.^1^, ^6^, ^7^, ^8^, ^9^ At present, chemotherapy, radiotherapy and surgery are still main therapy strategies for patients with ESCA, but the therapy efficacy is not very satisfactory, which may be because of the facts that most patients with ESCA was diagnosed in an advanced stage, coupled with the appearance of metastasis loci.^10^, ^11^ Therefore, it is in dire need of seeking for the new therapeutic strategy to improve the patients' prognosis.

Long non‐coding RNA (lncRNA) is a group of transcripts longer than 200 nucleotides, which can be split into 6 different types as follows: promoter‐associated transcripts, sense, antisense, bidirectional, intronic, intergenic and 3′UTR‐associated transcripts.^12^, ^13^ LncRNAs may be located in the cytoplasm or nucleus, but are mainly present in cell nucleus.^14^ LncRNAs are widely involved in the regulation of diverse biological processes.^15^, ^16^ In recent years, increasing evidence has revealed the important regulatory roles of lncRNAs in the occurrence and progression of ESCA.^17^ Notably, lncRNA‐miRNA‐mRNA regulatory axis widely participates in oesophageal carcinogenesis.^18^ Many lncRNAs play pivotal roles in maintaining and promoting the biological characteristics of tumour cells, and thus, lncRNAs may be the attractive therapeutic targets in a variety of tumours.^19^, ^20^ Our recent work identified many differential lncRNA through TCGA database, and we revealed that ZEB1‐AS1 was significantly up‐regulated in ESCA,^21^ but its precise functions remain unknown. It is a fact that ZEB1‐AS1 is closely correlated with tumour occurrence and development, such as bladder cancer,^22^ glioma,^23^ melanoma^24^ and non–small‐cell lung cancer,^25^ and these data suggest that ZEB1‐AS1 is involved in tumour progression via multiple different molecular mechanisms, suggesting the complexity of ZEB1‐AS1 function in these tumours. However, how ZEB1‐AS1 is regulated during ESCC development is still unclear, and therefore, herein, the expression pattern of ZEB1‐AS1 and its regulatory role in the proliferation and invasion ability of ESCC were investigated, which will propose ZEB1‐AS1/ZEB1 regulatory axis as an underlying therapeutic target for ESCC therapy.

## 2 MATERIALS AND METHODS

### 2.1 Tissue samples

Resected ESCC tissues and normal oesophageal epithelial tissues were collected from the First Affiliated Hospital of Zhengzhou University, Zhengzhou, Henan, China, including 56 ESCC samples and 56 paired normal samples, which was stored in liquid nitrogen. Informed consent of all tissue samples confirmed by pathologist was obtained from each participant. All patients did not receive any treatments prior surgery. The current study was authorized by the Institutional Research Ethics Committee of Zhengzhou University.

### 2.2 In situ hybridization (ISH) assay

Human ZEB1‐AS1 (GenBank accession number: NR_024284) probe with a 53 bp was obtained using ZEB1‐AS1 specific primers (F: 5′‐AGCCTCCTTAGTAGAGCGGA‐3′; R: 5′‐AAGTGAGACAAGCACCGTGT‐3′). The PCR product was labelled with digoxigenin (Promega Corporation) according to the manufacturer's instruction. ISH assay for ZEB1‐AS1 expression was carried out according to previous report.^26^ In brief, tissue sections were deparaffinized in xylene and graded alcohol, followed by heat treatment for 15 minutes in the buffer at 100°C. The tissues were treated using pepsin for 10 minutes at room temperature (RT), and then, ZEB1‐AS1 probe labelled with digoxigenin was added to the tissue sections. The tissue slides were denatured 96°C for 5 minutes and were placed in a moisturized chamber for hybridization reaction overnight at 37°C. Finally, NBT/BCIP was employed to develop the signal. PBS was used as a negative control instead of ZEB1‐AS1 probe.

### 2.3 Immunohistochemistry (IHC)

Immunohistochemistry assay was carried out according to previous document.^27^ Briefly, tumour tissues with paraffin embedding were serially cut with 4 μm, and the sections were dewaxed and rehydrated according to standard protocol. Subsequently, 3% H_2_O_2_ was employed to inactivate the endogenous peroxidase for 10 minutes, followed by heating the tissue sections in EDTA solution (1 mmol/L, pH 9.0) for antigen retrieval. The sections were rinsed using PBS buffer for 5 minutes and then blocked using 3% BSA solution for 30 minutes at RT. Afterwards, the sections were incubated with ZEB1 primary antibody (Abcam) with 1:200 dilution overnight at 4°C, and then, horseradish peroxidase (HRP)‐conjugated secondary antibody was added to sections for 30 minutes at RT. The staining signal was developed with a DAB kit (Zhongshan Golden Bridge Biotechnology Company).

### 2.4 Staining scores of ISH and IHC

All staining results were independently evaluated in a double‐blinded manner by two pathologists according to the following standards. The number of positive cells was scored as follows: 0 (no staining); 1 (0.01%‐25%); 2 (25.01%‐50%); 3 (50.01%‐75%); and 4 (>75%). The staining intensity was evaluated in the following: 0 (no signal); 1 (weak); 2 (moderate); and 3 (strong). The final result was obtained according to the following formula ‘the score of positive cell number × the score of staining intensity’ and was regarded as follows: 0 (negative, −); 1‐4 (weak, +); 5‐8 (moderate, ++); and 9‐12 (strong, +++). Score less than or equal to 4 was considered as low expression, and other score was high expression.

### 2.5 Real‐time quantitative PCR (qPCR)

Total RNA was extracted from ESCC tissues and cells, which was reverse transcripted to cDNA using lnRcute lncRNA cDNA first strand synthesis kit (Tiangen Biotech). qPCR (Tiangen Biotech) was employed to determine the ZEB1‐AS1 and ZEB1 expressions according to manufacturer's instruction using the primers as follows: ZEB1‐AS1: 5′‐GATGCCGGGAAACCGTAGG‐3′ and 5′‐CTACTAAGGAGGCTGCTGGC‐3′ (product size: 175bp), ZEB1: 5′‐GTGACGCAGTCTGGGTGTAA‐3′ and 5′‐TGAGTCCTGTTCTT GGTCGC‐3′ (product size: 229bp).

### 2.6 Cell culture and transfection

The ESCC cell lines (EC9706, TE1, Eca109, Kyse70 and Kyse450) and normal oesophageal epithelial cell Het‐1A kept in liquid nitrogen in our laboratory were cultured in RPMI‐1640 medium supplemented with 10% foetal bovine serum (FBS, Gibco Company), 100 μg/mL streptomycin (Sigma‐Aldrich) and 100 U/mL penicillin (Sigma‐Aldrich) in a humidified 5% CO_2_ incubator at 37°C. ZEB1‐AS1 siRNA (#1 sense: AACUUCUAGCCUCUCUUUCAA, antisense: GAAAGAGAGGCUAGAAGUUCC; #2 sense: UUUAGGAAGGAAUUCAUGGCC, antisense: CCAUGAAUUCCUUC CUAAAUG), negative control (NC): 5′‐UUCUCCGAACGUGUCACGUTT‐3′ (sense); 5′‐ACGUGACACGUUCGGAGAATT‐3′ (antisense), ZEB1 siRNA (Santa Cruz company), control siRNA (Santa Cruz company), pcDNA3.1 empty vector and pcDNA3.1‐ZEB1 (ZEB1) were transfected to EC9706 and TE1 cells by Lipofectamine™ 2000 (Invitrogen Life Technologies) according to manufacturer's instruction.

### 2.7 Methylation‐specific PCR (MSP)

Methylation‐specific PCR was performed according to previous report.^28^ The primer for MSP was designed using MethPrimer online software as described previously in the following^28^: Methylaion‐F: 5′‐TTTTTCGTTTGTGTTTAAATGTTC‐3′, Methylation‐R: 5′‐ ATATCGTAAAACCGAAAATATCGTA‐3′; Unmethylation‐F: 5′‐TTTTTGTTTGTGTTTAAATGTTTGA‐3′; Unmethylation‐R: 5′‐ATATCATAAAACCAAAAATATCAT A‐3′. Genomic DNA was obtained from ESCC tissues and cells using genomic DNA extract kit (TIANGEN), and PCR was performed according to manufacturer's instruction.

### 2.8 CCK‐8 experiment

EC9706 and TE1 cells at a density of 2000 cells/well were seeded into 96‐well plate, and then, these cells transfected with ZEB1‐AS1 siRNA (si‐ZEB1‐AS1), ZEB1 siRNA (si‐ZEB1), negative control (si‐NC) and pcDNA3.1‐ZEB1 (ZEB1) in triplicate were applied to corresponding well. Absorbance value (450 nm) was measured in a microplate reader (Thermo Scientific), and cell viability was measured by CCK‐8 kit (Beyotime Biotech) according to manufacturer's protocol.

### 2.9 Cell invasion assay

Cell invasion was investigated by Transwell chamber harbouring Matrigel (BD Biosciences). Briefly, EC9706 and TE1 cells (1 × 10^5^) were placed in the upper layer of chamber, and meanwhile, 20% FBS was added to underlayer of chamber. Subsequently, invasive cells were fixed using methanol, followed by staining with crystal violet 48 hours after transfection. Finally, the number of invasive cells was investigated under the field of 200× magnification.

### 2.10 Western blot

Total proteins were extracted from ESCC cells using RIPA lysis (Solarbio), and the concentration was measured by Bradford method. The proteins were separated by SDS‐PAGE and then transferred to PVDF membranes (Millipore Corporation). The primary antibodies against E‐cadherin, N‐cadherin, vimentin, ZEB1 and β‐actin (1:200 dilution, Abcam) were incubated with PVDF membrane (Roche) overnight at RT after blocking with skimmed milk. Subsequently, the secondary antibody (ZSGB‐BIO) was added to PVDF membrane. Finally, enhanced chemiluminescence reagents (Beyotime) were utilized to develop the protein signal.

### 2.11 Animal experiment

Female BALB/c nude mice with 4‐6 weeks old were purchased from Weitong Lihua Experimental Animal Technical Company. All mice were maintained in a pathogen‐free facility. EC9706 and TE1 cells (1 × 10^6^) harbouring stable ZEB1‐AS1 knockdown were subcutaneously inoculated into the back of nude mice. Once the tumour volumes reached approximate 100 mm^3^, the mice were randomly split into three groups: pLVX‐shRNA‐NC, pLVX‐shRNA‐ZEB1‐AS1 and pLVX‐shRNA‐ZEB1‐AS1 plus ZEB1. Tumour volumes were measured twice a week. When the measurement was terminated, tumour growth curve was made. All protocols were approved by the Institutional Committee for Use and Care of Laboratory Animals of Zhengzhou University.

### 2.12 Statistical assay

All data expressed as mean ± standard deviation (SD) were repeated in triplicate, which were examined using GraphPad Prism 6.0 software. The data regarding ISH and IHC were examined using chi‐square, and survival assay was performed using log‐rank test. The comparisons of two groups were determined using *t* test, and comparisons of three groups or above were investigated using one‐way ANOVA. A *P* value less than 0.05 were regarded to be significant.

## 3 RESULTS

### 3.1 ZEB1‐AS1 and ZEB1 levels in ESCC tissues and cells and their prognosis power in ESCC

**Figure 1:**
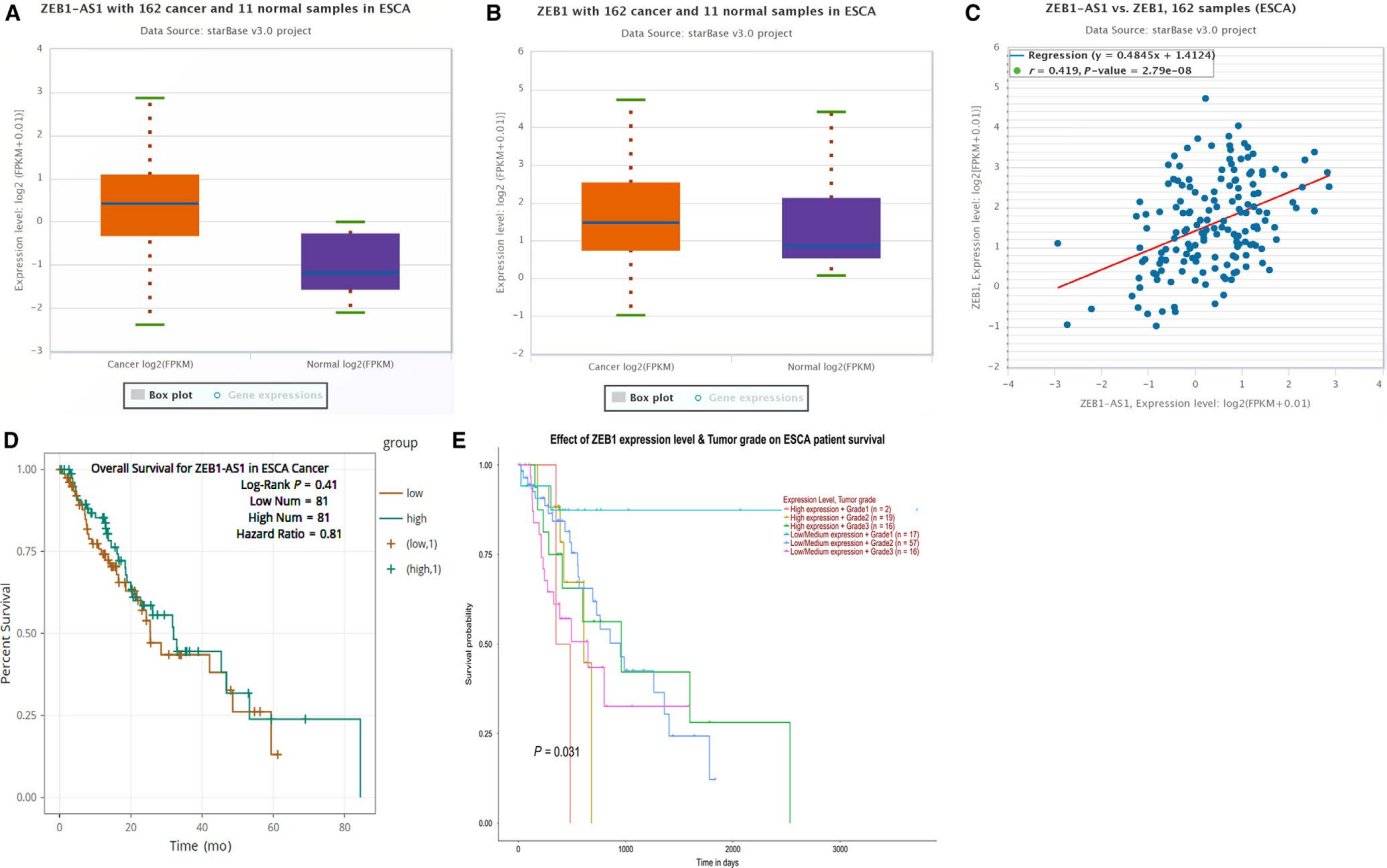
TCGA database assay for the expressions of ZEB1‐AS1 and ZEB1 as well as their prognosis in ESCA. A,B, starBase v3.0 assay for ZEB1‐AS1 and ZEB1 expressions in ESCA tissues and normal tissues; C, starBase v3.0 assay for co‐expression of ZEB1‐AS1 and ZEB1 in ESCA tissues; D, starBase v3.0 assay for the correlation of ZEB1‐AS1 with prognosis of the patients with ESCA; E, UALCAN analysis for the association of ZEB1 with prognosis of the patients with ESCA

**Figure 2:**
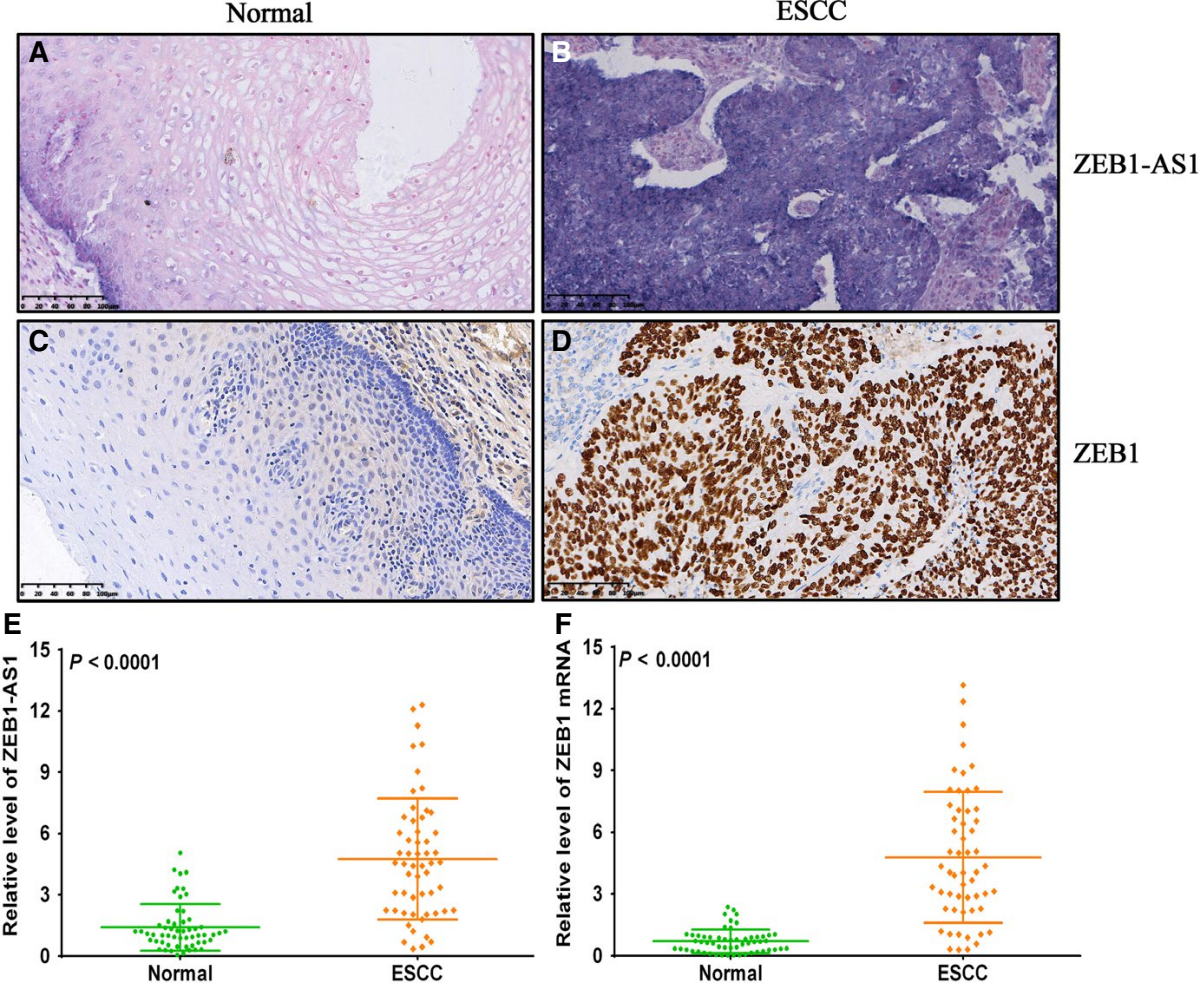
The expressions of ZEB1‐AS1 and ZEB1 in ESCC tissues and paired normal tissues. A, In situ hybridization detection for ZEB1‐AS1 level in normal oesophageal epithelial tissues, bar = 100 μm; B, In situ hybridization detection for ZEB1‐AS1 level in ESCC tissues, bar = 100 μm; C, Immunohistochemistry assay for ZEB1 protein expression in normal oesophageal epithelial tissues, bar = 100 μm; D, Immunohistochemistry assay for ZEB1 protein expression in ESCC tissues, bar = 100 μm; E, qPCR detection for ZEB1‐AS1 level in ESCC tissues and paired normal tissues; F, qPCR detection for ZEB1 mRNA level in ESCC tissues and paired normal tissues

TCGA database integrating UALCAN and starBase was employed to investigate the ZEB1‐AS1 and ZEB1 levels in ESCC tissues and its prognostic value. We unveiled that the levels of ZEB1‐AS1 and ZEB1 were up‐regulated in ESCA tissues (Figure 1A,B), and their expressions displayed markedly positive correlations in ESCA tissues (Figure 1C). Notably, ZEB1‐AS1 was not related to the prognosis of the patients with ESCA (Figure 1D), but the survival ratio of the patients with high ZEB1 level in different grade ESCA patients was lower than that with low ZEB1 level (*P* < .05) (Figure 1E). To validate these findings, qPCR, ISH and IHC were utilized to detect the levels of ZEB1‐AS1 and ZEB1 in 56 cases of ESCC tissues and para‐carcinoma tissues. The results of ISH and IHC demonstrated that ZEB1‐AS1 and ZEB1 expressions in ESCC tissues (positive ratio: 44.6% and 41.1%) were both higher than those in normal tissues (14.3% and 12.5%) (Figure 2A‐D), which were also confirmed by qPCR (Figure 2E,F). Our results herein imply that ZEB1‐AS1 and ZEB1 may play oncogenic role in ESCC.

### 3.2 The correlations of ZEB1‐AS1 and ZEB1 expressions with clinicopathological features in ESCC

To explore the possible biological role of ZEB1‐AS1 and ZEB1 in ESCC, SPSS 21.0 software was utilized to dissect the correlations of ZEB1‐AS1 and ZEB1 levels with clinicopathological factors, respectively. The results revealed that ZEB1‐AS1 and ZEB1 levels were both associated with lymph node metastasis and TNM staging (*P* < .01), but not correlated with patients' gender, age, invasion depth and histological grade (*P* > .05) (Table 1 and 2). These data imply that ZEB1‐AS1 and ZEB1 may exert pivotal role in the development and progression of ESCC.

**Table 1:** The correlation between ZEB1‐AS1 expression and clinicopathological features of ESCC

Characteristics	n	ZEB1‐AS1 expression		*X^2^*	*P* value
		Low	High		
Total cases	56	31	25		
Gender					
Male	38	19	19		
Female	18	12	6	1.373	0.241
Age					
≥60	33	17	16		
<60	23	14	9	0.480	0.488
Histological grade					
High	15	11	4		
Medium	19	12	7		
Poor	22	8	14	5.641	0.060
TNM staging					
I + II	26	22	4		
III + IV	30	9	21	16.812	0.000
Lymph node metastasis					
Yes	21	6	15		
No	35	25	10	9.755	0.002

**Table 2:** The correlation between ZEB1 expression and clinicopathological features of ESCC

Characteristics	n	ZEB1 expression		*X^2^*	*P* value
		Low	High		
Total cases	56	33	23		
Gender					
Male	38	20	18		
Female	18	13	5	1.937	0.164
Age					
≥60	33	17	16		
<60	23	16	7	1.825	0.177
Histological grade					
High	15	11	4		
Medium	19	13	6		
Poor	22	9	13	4.945	0.084
TNM staging					
I + II	26	23	3		
III + IV	30	10	20	17.490	0.000
Lymph node metastasis					
Yes	21	7	14		
No	35	26	9	9.095	0.003

### 3.3 ZEB1‐AS1 and ZEB1 are both correlated with TNM staging, lymph node metastasis and poor prognosis in ESCC

**Figure 3:**
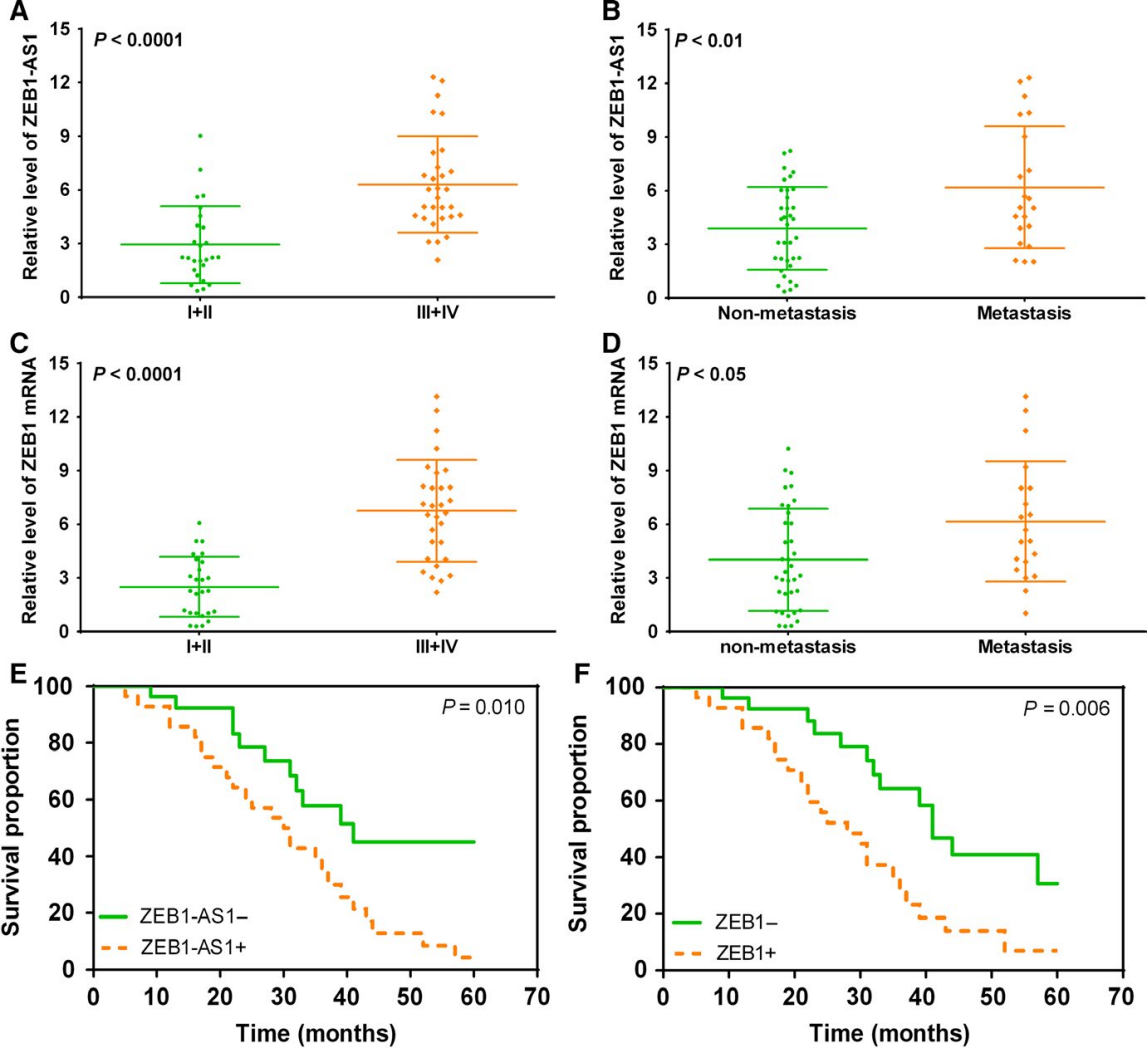
High ZEB1‐AS1 and ZEB1 levels predict higher TNM staging, lymph node metastasis and poor prognosis in patients with ESCC. A, qPCR detection for ZEB1‐AS1 level in ESCC patients with I + II and III + IV; B, qPCR assay for ZEB1‐AS1 level in ESCC patients with and without lymph node metastasis; C, qPCR detection for ZEB1 in ESCC patients with I + II and III + IV; D, qPCR assay for ZEB1 level in ESCC patients with and without lymph node metastasis; E, high ZEB1‐AS1 level predicts poor prognosis in patients with ESCC; F, high ZEB1 level predicts poor prognosis in patients with ESCC

To further explore the underlying role of ZEB1‐AS1 and ZEB1 in TNM staging, lymph node metastasis and prognosis in ESCC, qRT‐PCR was used to analyse the associations of ZEB1‐AS1 and ZEB1 with TNM staging, lymph node metastasis and prognosis in ESCC. We found that ZEB1‐AS1 levels in ESCC patients with III + IV staging and lymph node metastasis were markedly higher than those with I + II staging and without lymph node metastasis (Figure 3A,B), and similar results were found in ZEB1 expression pattern (Figure 3C,D). Most importantly, high ZEB1‐AS1 and ZEB1 levels both predicted poor prognosis of patients with ESCC (Figure 3E,F).

### 3.4 ZEB1‐AS1 promoter hypomethylation promotes ZEB1‐AS1 overexpression in ESCC

**Figure 4:**
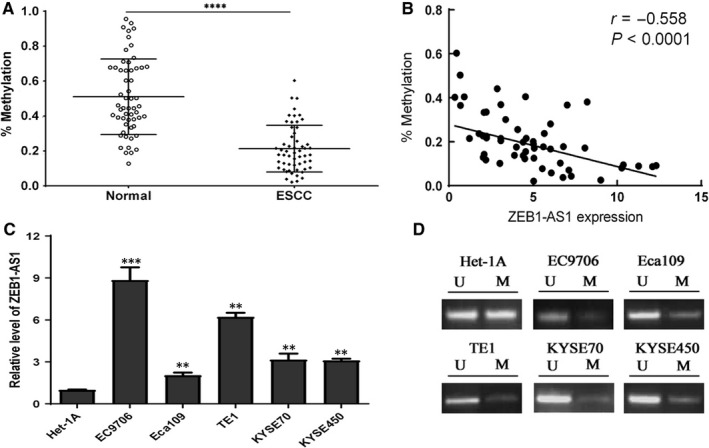
The hypomethylation of ZEB1‐AS1 promoter is tightly correlated with ZEB1‐AS1 up‐regulation in ESCC tissues and cells. A, MSP assay for methylation level of ZEB1‐AS1 promoter in ESCC tissues and normal tissues; B, a negative correlation between ZEB1‐AS1 promoter methylation and ZEB1‐AS1 expression level; C, qPCR detection for ZEB1‐AS1 level in different ESCC cells (EC9706, Eca109, TE1, Kyse70 and Kyse450) and normal oesophageal epithelial cell Het‐1A; D, MSP detection for methylation status of ZEB1‐AS1 promoter in ESCC cells and normal oesophageal epithelial cell Het‐1A

To elucidate the underlying factors regarding ZEB1‐AS1 overexpression in ESCC, MSP was utilized to examine the methylation status of ZEB1‐AS1 promoter in ESCC tissues and cells. Our results demonstrated that methylation level of ZEB1‐AS1 promoter in ESCC tissues was obviously lower than that in normal tissues (*P* < .0001) (Figure 4A), and a negative correlation between methylation level of ZEB1‐AS1 promoter and ZEB1‐AS1 expression was found (Figure 4B). Subsequent investigation uncovered that the relative levels of ZEB1‐AS1 in ESCC cells (EC9706, Eca109, TE1, Kyse70 and Kyse450) were markedly higher than those in Het‐1A cell (*P* < .01), in which EC9706 and TE1 cells exhibited the highest ZEB1‐AS1 level (Figure 4C). The results from different oesophageal cells revealed that methylation level of ZEB1‐AS1 promoter in different ESCC cells was evidently lower than that in Het‐1A cell (Figure 4D). These data imply that ZEB1‐AS1 at high level may be tightly associated with ZEB1‐AS1 promoter hypomethylation.

### 3.5 ZEB1‐AS1 down‐regulation suppresses proliferation and invasion ability in ESCC

**Figure 5:**
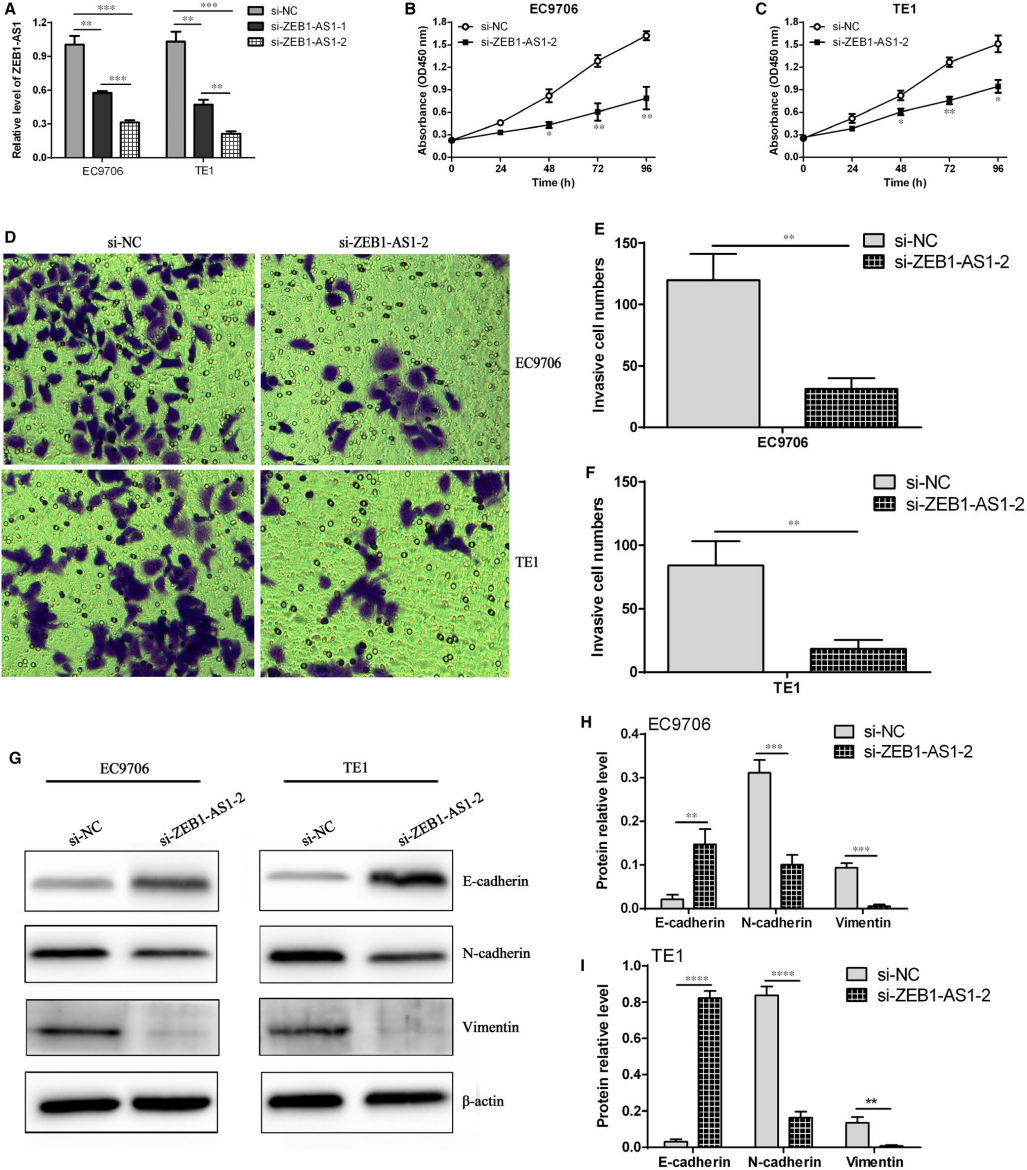
ZEB1‐AS1 down‐regulation contributed to the inhibition of cell proliferation and invasion in ESCC cells. A, qPCR detection of ZEB1‐AS1 expression in EC9706 and TE1 cells after transfection with si‐NC and si‐ZEB1‐AS1; B and C, CCK‐8 assay for cell proliferation after transfection with si‐NC and si‐ZEB1‐AS1‐2, ^*^ *P* < .05 and ^**^ *P* < .01, compared with si‐NC group; D, transwell chamber assay for cell invasion after transfection with si‐NC and si‐ZEB1‐AS1‐2; E and F, statistical assay for invasive cell number in EC9706 and TE1 cells, ^**^ *P* < .01, compared with si‐NC group; G, Western blot assay for the expressions of E‐cadherin, N‐cadherin and vimentin proteins in different treatment EC9706 and TE1 cells; H and I, the relative expression of E‐cadherin, N‐cadherin and vimentin proteins in different treatment EC9706 and TE1 cells, ^**^ *P* < .01, ^***^ *P* < .001 and ^****^ *P* < .0001, compared with si‐NC group

To preliminarily dissect ZEB1‐AS1 functions in ESCC, the effects of ZEB1‐AS1 siRNA on cell proliferation and invasion ability of ESCC cells were investigated. The results revealed that si‐ZEB1‐AS1‐1 and si‐ZEB1‐AS1‐2 dramatically reduced ZEB1‐AS1 levels in EC9706 and TE1 cells, compared with si‐NC (*P* < .01), and the interfering efficacy of si‐ZEB1‐AS1‐2 was obviously better than that of si‐ZEB1‐AS1‐1 (Figure 5A). CCK‐8 experiment demonstrated that si‐ZEB1‐AS1‐2 evidently suppressed cell proliferation in EC9706 and TE1 cells, compared with si‐NC group (Figure 5B,C). Further investigation revealed that ZEB1‐AS1 down‐regulation markedly inhibited cell invasion ability in EC9706 and TE1 cells (Figure 5D,E,F). Mechanically, ZEB1‐AS1 down‐regulation significantly increased the E‐cadherin level and reduced the N‐cadherin and vimentin levels in EC9706 and TE1 cells (Figure 5G,H,I). These findings imply that ZEB1‐AS1 may play an important role in cell proliferation and invasion in ESCC cells.

### 3.6 ZEB1 siRNA markedly suppresses the proliferation and invasion ability in ESCC

**Figure 6:**
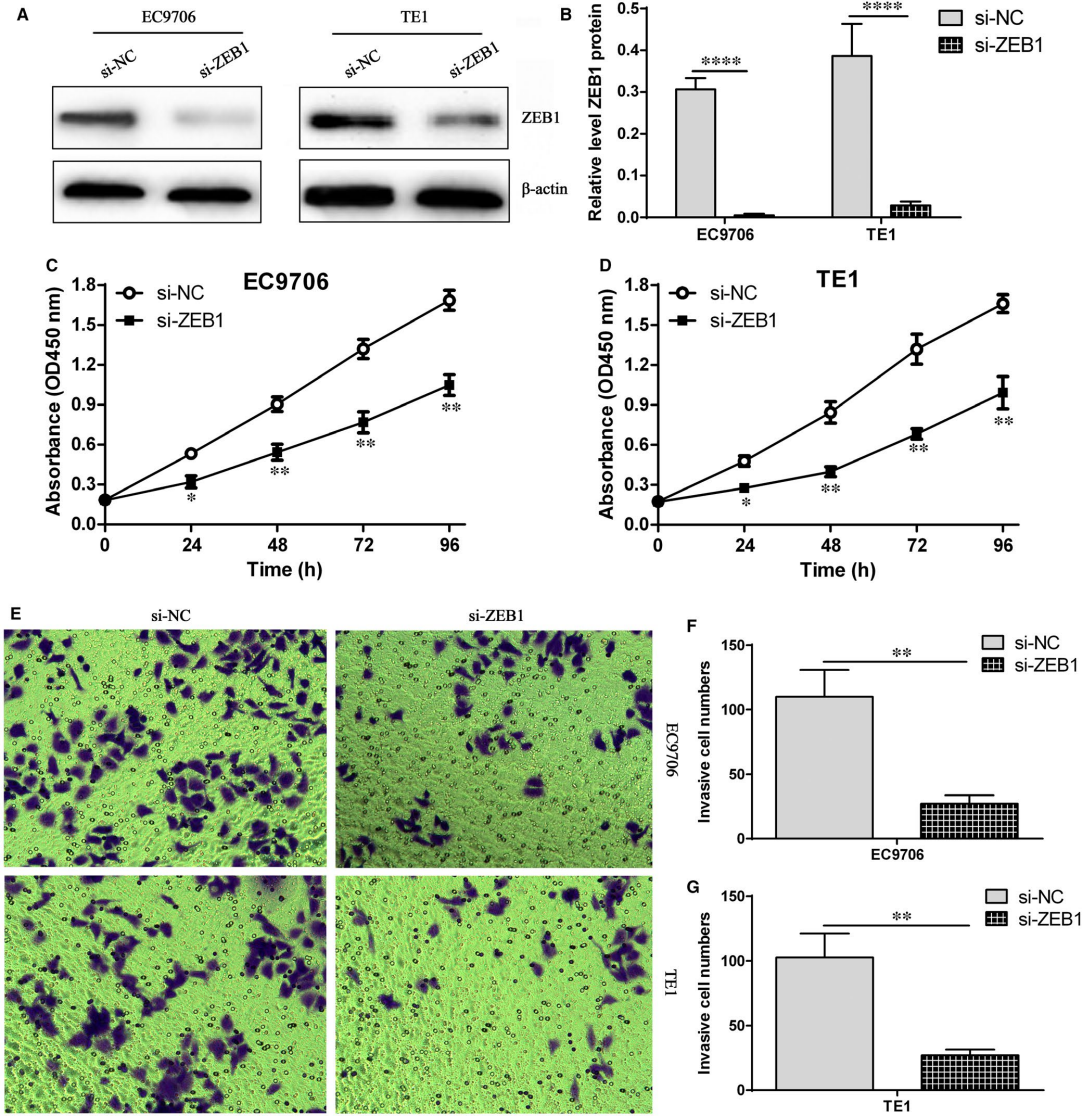
ZEB1 down‐regulation elicited the inhibition of proliferation and invasion in ESCC cells. A, ZEB1 siRNA markedly reduced ZEB1 protein expression in EC9706 and TE1 cells; B, relative level of ZEB1 protein in EC9706 and TE1 cells, ^****^ *P* < .0001, compared with si‐NC group; C, CCK‐8 experiment assay for cell proliferation in si‐NC group and si‐ZEB1 group in EC9706 cells, ^*^ *P* < .05 and ^**^ *P* < .01, compared with si‐NC group; D, CCK‐8 experiment assay for cell proliferation in si‐NC group and si‐ZEB1 group in TE1 cells, ^*^ *P* < .05 and ^**^ *P* < .01, compared with si‐NC group; E, transwell chamber investigation for cell invasion ability in EC9706 and TE1 cells; F, statistical assay for invasive cell number in si‐NC group and si‐ZEB1 group in EC9706 cells, ^**^ *P* < .01, compared with si‐NC group; G, statistical assay for invasive cell number in si‐NC group and si‐ZEB1 group in TE1 cells, ^**^ *P* < .01, compared with si‐NC group

To further elucidate whether ZEB1 also exerts a pivotal role in proliferation and invasion of ESCC, we detected the effect of ZEB1 siRNA on proliferation and invasion ability in ESCC cells. We found ZEB1 siRNA significantly suppressed ZEB1 expression in EC9706 and TE1 cells (Figure 6A,B). Further CCK‐8 experiment revealed that ZEB1 down‐regulation contributed to proliferation inhibition in EC9706 and TE1 cells (Figure 6C,D). Besides, ZEB1 down‐regulation markedly inhibited invasion ability in EC9706 and TE1 cells (Figure 6E,F,G). Our data indicate that ZEB1 may exert the crucial regulatory role in cell proliferation and invasion in ESCC cells.

### 3.7 ZEB1 overexpression reverses the inhibitory effect of proliferation and invasion mediated by ZEB1‐AS1 siRNA in ESCC cells

**Figure 7:**
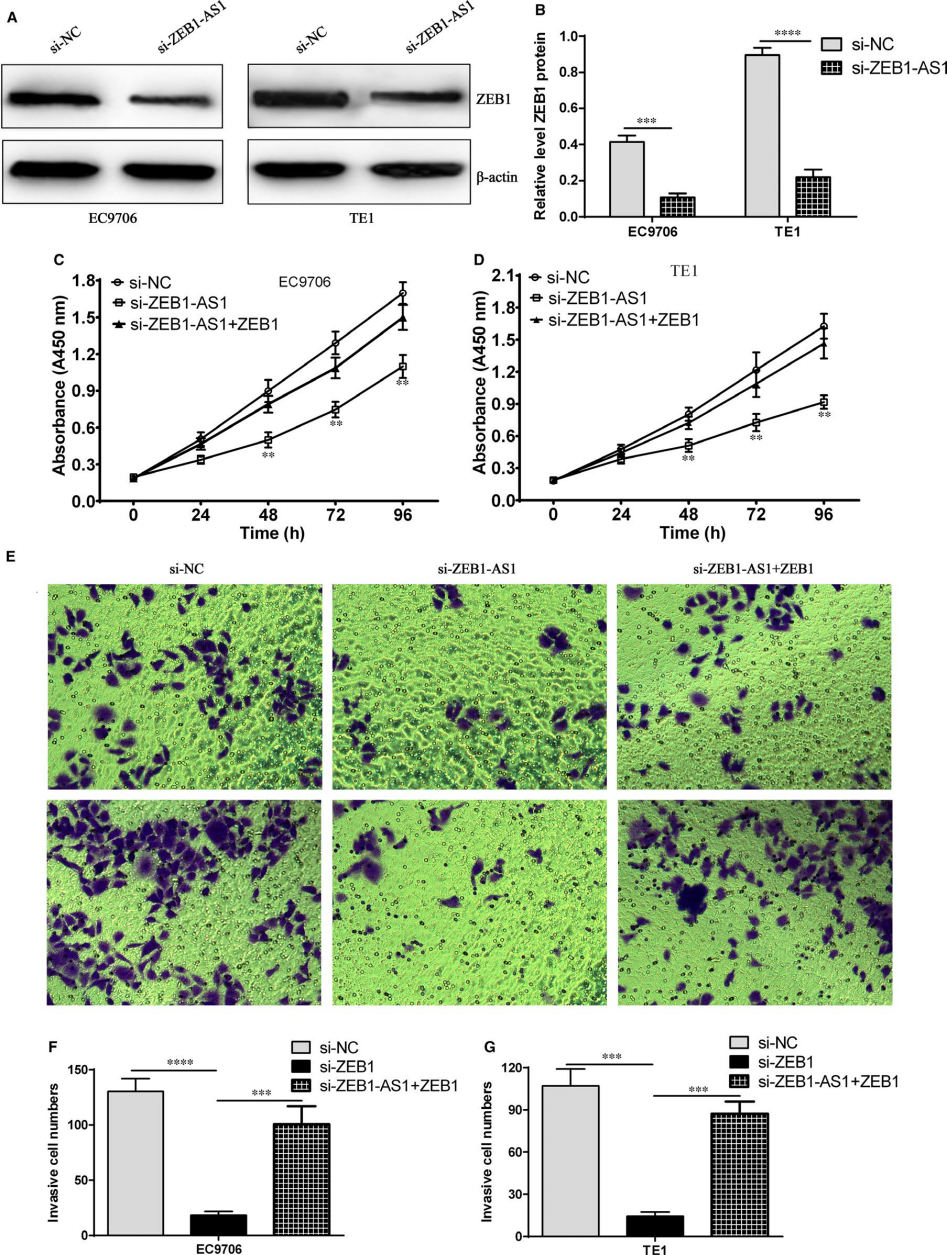
ZEB1‐AS1 suppresses cell proliferation and invasion by targeting ZEB1. A, ZEB1 protein level was evaluated by Western blot after transfection with si‐ZEB1‐AS1 in EC9706 and TE1 cells; B, Relative level of ZEB1 protein after transfection with si‐ZEB1‐AS1 in EC9706 and TE1 cells, ^***^ *P* < .001 and ^****^ *P* < .0001, compared with si‐NC group; C: CCK‐8 experiment assay for cell proliferation in si‐NC group, si‐ZEB1‐AS1 and si‐ZEB1‐AS1 plus ZEB1 overexpression group in EC9706 cells, ^**^ *P* < .01, compared with si‐NC group; D: CCK‐8 experiment assay for cell proliferation in si‐NC group, si‐ZEB1‐AS1 and si‐ZEB1‐AS1 plus ZEB1 overexpression group in TE1 cells, ^**^ *P* < .01, compared with si‐NC group; E, transwell chamber assay for cell invasion ability in si‐NC group, si‐ZEB1‐AS1 and si‐ZEB1‐AS1 plus ZEB1 overexpression group in EC9706 and TE1 cells; F, statistical assay for invasive cell number in si‐NC group, si‐ZEB1‐AS1 and si‐ZEB1‐AS1 plus ZEB1 overexpression group in EC9706 cells, ^***^ *P* < .001 and ^****^ *P* < .0001, compared with si‐NC group; G, statistical assay for invasive cell number in si‐NC group, si‐ZEB1‐AS1 and si‐ZEB1‐AS1 plus ZEB1 overexpression group in TE1 cells, ^***^ *P* < .001, compared with si‐NC group

It is well documented that antisense lncRNAs control the level of the sense genes directly or indirectly.^29^ ZEB1‐AS1 is an antisense cognate gene of ZEB1, and we put forward that whether ZEB1‐AS1 can regulate ZEB1 expression, whereas whether ZEB1 overexpression can reverse the biological process mediated by ZEB1‐AS1 down‐regulation in ESCC cells. Therefore, the effect of ZEB1‐AS1 on ZEB1 expression and the biological effect triggered by ZEB1 overexpression were detected. The results revealed that si‐ZEB1‐AS1 markedly down‐regulated ZEB1 protein level in EC9706 and TE1 cells (Figure 7A,B). Further investigation showed that si‐ZEB1‐AS1 strikingly restrained cell proliferation and invasion ability, whereas ZEB1 overexpression obviously reversed the inhibitory effect of proliferation and invasion elicited by si‐ZEB1‐AS1 (Figure 7C‐G), suggesting ZEB1‐AS1 plays a vital regulatory role in cell proliferation and invasion by manipulating ZEB1 expression in ESCC cells.

### 3.8 ZEB1‐AS1 down‐regulation suppresses tumour growth in ESCC cell xenografted nude mice

**Figure 8:**
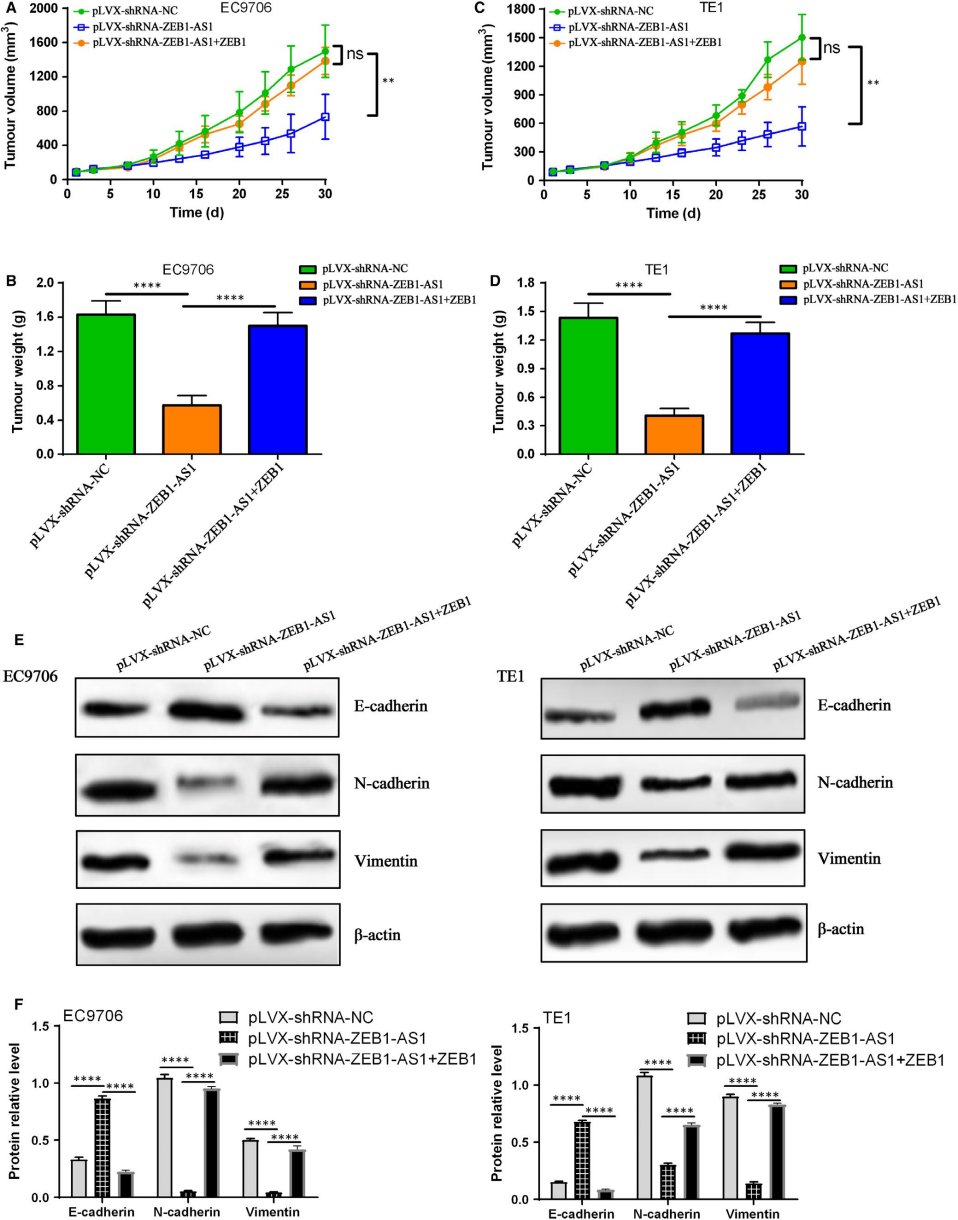
ZEB1‐AS1 down‐regulation suppresses tumour growth in EC9706 and TE1 xenografted nude mice. A, ZEB1‐AS1 down‐regulation suppressed tumour growth in EC9706 xenografted nude mice, whereas ZEB1 overexpression partly reversed the inhibitory efficacy, ns indicates no significance, ^**^ *P* < .01, compared with pLVX‐shRNA‐NC group and pLVX‐shRNA‐ZEB1‐AS1 plus ZEB1; B, Tumour weight in different treatment group, ^****^ *P* < .0001, compared with pLVX‐shRNA‐NC group and pLVX‐shRNA‐ZEB1‐AS1 plus ZEB1; C, ZEB1‐AS1 down‐regulation suppression tumour growth in TE1 xenografted nude mice, whereas ZEB1 overexpression partly reversed the inhibitory efficacy, ns indicates no significance, ^**^ *P* < .01, compared with pLVX‐shRNA‐NC group and pLVX‐shRNA‐ZEB1‐AS1 plus ZEB1; D, Tumour weight in different treatment group, ^****^ *P* < .0001, compared with pLVX‐shRNA‐NC group and pLVX‐shRNA‐ZEB1‐AS1 plus ZEB1; E, Western blot assay for E‐cadherin, N‐cadherin and vimentin protein experssions in EC9706 and TE1 xenografted nude mice, and β‐actin was used for loading control; F, relative levels of E‐cadherin, N‐cadherin and vimentin protein experssions in EC9706 and TE1 xenografted nude mice, ^****^ *P* < .0001, compared with pLVX‐shRNA‐ZEB1‐AS1

To explore the potential role of ZEB1‐AS1 in tumorigenesis in ESCC cells xenografted nude mice, ESCC EC9706 and TE1 cells stably expressing shRNA‐ZEB1‐AS1 or shRNA‐NC were subcutaneously inoculated into the back of nude mice, and tumour volume and weight were measured. The results indicated that ZEB1‐AS1 down‐regulation evidently suppressed tumour growth and reduced tumour weight, whereas ZEB1 overexpression partly recovered the inhibitory effect mediated by pLVX‐shRNA‐ZEB1‐AS1 in ESCC EC9706 and TE1 cells xenografted nude mice (Figure 8A‐D). Further investigation revealed that pLVX‐shRNA‐ZEB1‐AS1 markedly increased E‐cadherin level and decreased the levels of N‐cadherin and vimentin proteins in EC9706 and TE1 cells xenografted nude mice, which was partly reversed by ZEB1 overexpression (Figure 8E,F). Our data herein imply that ZEB1‐AS1 suppresses tumour growth by inhibiting ZEB1 level in ESCC.

## 4 DISCUSSION

In the current study, ZEB1‐AS1 functioned as a regulator of ZEB1 gene in ESCC, which was tightly associated with the regulation of proliferation and invasion of ESCC cells. Our data demonstrated that ZEB1‐AS1 and its cognate gene ZEB1 were both unregulated in ESCC tissues, and the up‐regulation of ZEB1‐AS1 and ZEB1 were both associated with TNM staging, lymph node metastasis and poor prognosis of patients with ESCC. Notably, hypomethylation of ZEB1‐AS1 promoter promoted the overexpression of ZEB1‐AS1 in ESCC tissues and cells. Further investigation revealed that ZEB1‐AS1 down‐regulation markedly suppressed cell proliferation and invasion ability of ESCC cells, coupled with EMT suppression, and meanwhile, ZEB1 down‐regulation significantly inhibited cell proliferation and invasion ability of ESCC cells. Most importantly, ZEB1‐AS1 down‐regulation obviously reduced ZEB1 level in ESCC cells, whereas ZEB1 overexpression reversed the suppression of proliferation and invasion elicited by ZEB1‐AS1 down‐regulation. Thus, our data presented herein reveal that ZEB1‐AS1/ZEB1 regulatory axis is implicated in the proliferation and invasion ability in ESCC and targeting ZEB1‐AS1‐ZEB1 regulatory axis may be a new target for therapy of patients with ESCC.

LncRNAs function as tumour suppressor or oncogene in a large number of tumours,^30^, ^31^ which may depend on tumour types and environment, and detailed elucidation of expression patterns and functions of lncRNAs will provide the early diagnostic biomarkers, prognostic determination and therapeutic target in many tumours. Increasing evidence has demonstrated that lncRNAs have become a potential diagnostic and prognostic marker in a variety of tumours.^32^, ^33^, ^34^ Therefore, the identification of novel lncRNAs implicated in the development and progression of ESCC will provide new strategy for better diagnosis and therapy for patients with ESCC. Several studies have revealed that ZEB1‐AS1 was frequently overexpressed in many tumours and was tightly associated with poor prognosis of patients with tumour. Chai H et al confirmed that ZEB1‐AS1 was significantly elevated, and its expression was correlated with tumour, nodes, metastases stage IV, loss of E‐cadherin expression and poor prognosis in gastric cancer.^35^ The data from meta‐analysis revealed that high ZEB1‐AS1 level was closely correlated with overall survival (HR =  2.16, 95% CI: 1.89‐2.47), poor histological grade, high tumour staging and lymph node metastasis among patients with cancer,^36^ which was similar with previous report.^37^ These findings indicate that suppression of ZEB1‐AS1 expression may be a potential strategy for therapy of many tumour patients. Here, we found ZEB1‐AS1 and ZEB1 expressions in ESCC tissues were both higher than those in normal tissues, and their high levels were both associated with TNM staging as well as lymph node metastasis (*P* < .01). More importantly, high ZEB1‐AS1 and ZEB1 levels both predicted poor prognosis of patients with ESCC. These data suggest that ZEB1‐AS1 and ZEB1 may participate in the progression and metastasis of ESCC. To elucidate the underlying factors regarding ZEB1‐AS1 overexpression in ESCC, the methylation status of ZEB1‐AS1 promoter in ESCC tissues and cells was detected in this study. We found that methylation level of ZEB1‐AS1 promoter in ESCC tissues and cells was significantly lower than that in normal tissues and cells (*P* < .0001), and meanwhile, a negative correlation between methylation level of ZEB1‐AS1 promoter and ZEB1‐AS1 expression was found, which is consistent with previous investigation in hepatocellular carcinoma.^28^ These data imply that ZEB1‐AS1 at high level may be tightly associated with ZEB1‐AS1 promoter hypomethylation.

Sustaining proliferative signalling and activating invasion and metastasis have been verified to be two main characteristics of tumours,^38^ and combined targeting two hallmarks may be a novel strategy for therapy of tumour patients. Cheng R et al revealed that ZEB1‐AS1 depletion suppressed the growth, migration, invasion and EMT in cervical cancer, which may be achieved by inhibiting ZEB1 expression.^39^ Moreover, ZEB1‐AS1 depletion markedly restrained the proliferation and induced apoptosis in colorectal cancer, whereas ZEB1‐AS1 elevation elicited the opposite effect.^40^ A recent investigation demonstrated that ZEB1‐AS1 silencing triggered cell proliferation suppression and decreases in the cell number in S phase in liver cancer by targeting miR‐365a‐3p.^41^ To further dissect the underlying role of ZEB1‐AS1 in ESCC, we firstly investigated the expression of ZEB1‐AS1 in ESCC cells. The data revealed that the relative levels of ZEB1‐AS1 in ESCC cells (EC9706, Eca109, TE1, Kyse70 and Kyse450) were dramatically higher than those in Het‐1A cell (*P* < .01), in which EC9706 and TE1 cells exhibited the highest ZEB1‐AS1 level. Further investigation revealed that ZEB1‐AS1 down‐regulation markedly blocked the proliferation and invasion ability, coupled with elevation of E‐cadherin expression and reduces of N‐cadherin and vimentin expressions in ESCC cells. Meanwhile, ZEB1 knockdown triggered the suppression of the proliferation and invasion ability in ESCC cells. Most importantly, ZEB1‐AS1 down‐regulation markedly suppressed ZEB1 expression, whereas ZEB1 overexpression reversed the biological effect mediated by ZEB1‐AS1 down‐regulation in vitro and in vivo, which was tightly associated with the changes in EMT markers including E‐cadherin, N‐cadherin and vimentin, suggesting ZEB1‐AS1 functions as oncogene by regulating ZEB1 expression in ESCC cells. Our data presented herein imply that ZEB1‐AS1 may be a vital regulator in proliferation and invasion of ESCC cells, and targeting ZEB1‐AS1 may be a novel strategy for therapy of patients with ESCC.

In conclusion, ZEB1‐AS1 and ZEB1 are both implicated in the development and progression of ESCC, and their overexpression is tightly related to TNM staging, lymph node metastasis and poor prognosis of patients with ESCC. Notably, hypomethylation of ZEB1‐AS1 promoter promoted the overexpression of ZEB1‐AS1 in ESCC tissues and cells, and ZEB1‐AS1 and ZEB1 are both implicated in proliferation and invasion ability in ESCC. Therefore, targeting ZEB1‐AS1/ZEB1 regulatory axis may be a novel strategy for therapy of patients with ESCC.

## CONFLICT OF INTEREST

The authors declare no conflict of interest.

## AUTHOR CONTRIBUTION

Yan Zhao and Shujun Yang: designed the whole experiment, dissected the data and prepared for the manuscript. Yan Zhao, Ning Wang, Xiaoshan Zhang and Hongtao Liu: performed the experiment, obtained the data and reviewed the manuscript.

## Data Availability

The data that support the findings of this study are available from the corresponding author upon reasonable request.

## References

[1] Pennathur A , Gibson MK , Jobe BA , Luketich JD . Oesophageal carcinoma. Lancet. 2013;381:400‐412. 23374478 10.1016/S0140-6736(12)60643-6

[2] Torre LA , Bray F , Siegel RL , Ferlay J , Lortet‐Tieulent J , Jemal A . Global cancer statistics, 2012. CA A Cancer J Clin. 2015;65(2):87‐108. 10.3322/caac.21262 25651787

[3] Arnold M , Soerjomataram I , Ferlay J , Forman D . Global incidence of oesophageal cancer by histological subtype in 2012. Gut. 2015;64:381‐387. 25320104 10.1136/gutjnl-2014-308124

[4] Conteduca V , Sansonno D , Ingravallo G , et al. Barrett's esophagus and esophageal cancer: an overview. Int J Oncol. 2012;41:414‐424. 22615011 10.3892/ijo.2012.1481

[5] Wang GQ , Jiao GG , Chang FB , et al. Long‐term results of operation for 420 patients with early squamous cell esophageal carcinoma discovered by screening. Ann Thorac Surg. 2004;77:1740‐1744. 15111177 10.1016/j.athoracsur.2003.10.098

[6] Law S , Kwong DL , Kwok KF , et al. Improvement in treatment results and long‐term survival of patients with esophageal cancer: impact of chemoradiation and change in treatment strategy. Ann Surg. 2003;238:339‐347; discussion 47–8. 14501500 10.1097/01.sla.0000086545.45918.ee PMC1422701

[7] Jemal A , Siegel R , Xu J , Ward E . Cancer Statistics, 2010. CA A Cancer J Clin. 2010;60(5):277‐300. 10.3322/caac.20073 20610543

[8] Sjoquist KM , Burmeister BH , Smithers BM , et al. Survival after neoadjuvant chemotherapy or chemoradiotherapy for resectable oesophageal carcinoma: an updated meta‐analysis. Lancet Oncol. 2011;12:681‐692. 21684205 10.1016/S1470-2045(11)70142-5

[9] Berger AC , Farma J , Scott WJ , et al. Complete response to neoadjuvant chemoradiotherapy in esophageal carcinoma is associated with significantly improved survival. J Clin Oncol. 2005;23:4330‐4337. 15781882 10.1200/JCO.2005.05.017

[10] Oppedijk V , van der Gaast A , van Lanschot JJ , et al. Patterns of recurrence after surgery alone versus preoperative chemoradiotherapy and surgery in the CROSS trials. J Clin Oncol. 2014;32:385‐391. 24419108 10.1200/JCO.2013.51.2186

[11] Shapiro J , van Lanschot J , Hulshof M , et al. Neoadjuvant chemoradiotherapy plus surgery versus surgery alone for oesophageal or junctional cancer (CROSS): long‐term results of a randomised controlled trial. Lancet Oncol. 2015;16:1090‐1098. 26254683 10.1016/S1470-2045(15)00040-6

[12] Ponting CP , Oliver PL , Reik W . Evolution and functions of long noncoding RNAs. Cell. 2009;136:629‐641. 19239885 10.1016/j.cell.2009.02.006

[13] Mercer TR , Dinger ME , Mattick JS . Long non‐coding RNAs: insights into functions. Nat Rev Genet. 2009;10:155‐159. 19188922 10.1038/nrg2521

[14] Djebali S , Davis CA , Merkel A , et al. Landscape of transcription in human cells. Nature. 2012;489:101‐108. 22955620 10.1038/nature11233 PMC3684276

[15] Liu B , Ye B , Yang L , et al. Long noncoding RNA lncKdm2b is required for ILC3 maintenance by initiation of Zfp292 expression. Nat Immunol. 2017;18:499‐508. 28319097 10.1038/ni.3712

[16] Ye B , Liu B , Yang L , et al. LncKdm2b controls self‐renewal of embryonic stem cells via activating expression of transcription factor Zbtb3. EMBO J. 2018;37(8):pii: e97174. 10.15252/embj.201797174 PMC5897780 29535137

[17] Huang X , Zhou X , Hu Q , et al. Advances in esophageal cancer: A new perspective on pathogenesis associated with long non‐coding RNAs. Cancer Lett. 2018;413:94‐101. 29104147 10.1016/j.canlet.2017.10.046

[18] Xue WH , Fan ZR , Li LF , et al. Construction of an oesophageal cancer‐specific ceRNA network based on miRNA, lncRNA, and mRNA expression data. World J Gastroenterol. 2018;24:23‐34. 29358879 10.3748/wjg.v24.i1.23 PMC5757122

[19] Botti G , Marra L , Malzone MG , et al. LncRNA HOTAIR as prognostic circulating marker and potential therapeutic target in patients with tumor diseases. Curr Drug Targets. 2017;18:27‐34. 26648066 10.2174/1389450117666151209122950

[20] Fu LL , Li CJ , Xu Y , et al. Role of lncRNAs as novel biomarkers and therapeutic targets in ovarian cancer. Crit Rev Eukaryot Gene Expr. 2017;27:183‐195. 28845767 10.1615/CritRevEukaryotGeneExpr.2017019244

[21] Liu H , Zhang Q , Lou Q , et al. Differential analysis of lncRNA, miRNA and mRNA expression profiles and the prognostic value of lncRNA in esophageal cancer. Pathol Oncol Res. 2019 10.1007/s12253-019-00655-8 [Epub ahead of print]. 30972633

[22] Gao R , Zhang N , Yang J , et al. Long non‐coding RNA ZEB1‐AS1 regulates miR‐200b/FSCN1 signaling and enhances migration and invasion induced by TGF‐beta1 in bladder cancer cells. J Exp Clin Cancer Res. 2019;38:111. 30823924 10.1186/s13046-019-1102-6 PMC6397446

[23] Meng L , Ma P , Cai R , Guan Q , Wang M , Jin B . Long noncoding RNA ZEB1‐AS1 promotes the tumorigenesis of glioma cancer cells by modulating the miR‐200c/141‐ZEB1 axis. Am J Transl Res. 2018;10:3395‐3412. 30662595 PMC6291700

[24] Wang Q , Zhang R , Liu D . Long non‐coding RNA ZEB1‐AS1 indicates poor prognosis and promotes melanoma progression through targeting miR‐1224‐5p. Exp Ther Med. 2019;17:857‐862. 30651872 10.3892/etm.2018.7005 PMC6307420

[25] Jin J , Wang H , Si J , Ni R , Liu Y , Wang J . ZEB1‐AS1 is associated with poor prognosis in non‐small‐cell lung cancer and influences cell migration and apoptosis by repressing ID1. Clin Sci. 2019;133:381‐392. 30626729 10.1042/CS20180983

[26] Liu HT , Wang N , Wang X , Li SL . Overexpression of Pim‐1 is associated with poor prognosis in patients with esophageal squamous cell carcinoma. J Surg Oncol. 2010;102:683‐688. 20544717 10.1002/jso.21627

[27] Dong J , Wang R , Ren G , et al. HMGA2‐FOXL2 axis regulates metastases and epithelial‐to‐mesenchymal transition of chemoresistant gastric cancer. Clin Cancer Res. 2017;23:3461‐3473. 28119367 10.1158/1078-0432.CCR-16-2180

[28] Li T , Xie J , Shen C , et al. Upregulation of long noncoding RNA ZEB1‐AS1 promotes tumor metastasis and predicts poor prognosis in hepatocellular carcinoma. Oncogene. 2016;35:1575‐1584. 26073087 10.1038/onc.2015.223

[29] Wang YQ , Jiang DM , Hu SS , et al. SATB2‐AS1 suppresses colorectal carcinoma aggressiveness by inhibiting SATB2‐dependent Snail transcription and epithelial‐mesenchymal transition. Cancer Res. 2019;79:3542‐3556. 30858153 10.1158/0008-5472.CAN-18-2900

[30] Wang Y , Li W , Chen X , Li Y , Wen P , Xu F . MIR210HG predicts poor prognosis and functions as an oncogenic lncRNA in hepatocellular carcinoma. Biomed Pharmacother. 2019;111:1297‐1301. 30841443 10.1016/j.biopha.2018.12.134

[31] Zhang J , Li Z , Liu L , et al. Long noncoding RNA TSLNC8 is a tumor suppressor that inactivates the interleukin‐6/STAT3 signaling pathway. Hepatology. 2018;67:171‐187. 28746790 10.1002/hep.29405

[32] Ma PJ , Guan QK , Xu DW , Zhao J , Qin N , Jin BZ . LncRNA PANDAR as a prognostic marker in Chinese cancer. Clin Chim Acta. 2017;475:172‐177. 29066211 10.1016/j.cca.2017.10.020

[33] Ye G , Guo L , Xing Y , Sun W , Yuan M . Identification of prognostic biomarkers of prostate cancer with long non‐coding RNA‐mediated competitive endogenous RNA network. Exp Ther Med. 2019;17:3035‐3040. 30906477 10.3892/etm.2019.7277 PMC6425256

[34] Ji B , Huang Y , Gu T , Zhang L , Li G , Zhang C . Potential diagnostic and prognostic value of plasma long noncoding RNA LINC00086 and miR‐214 expression in gastric cancer. Cancer Biomark. 2019;24:249‐255. 30689553 10.3233/CBM-181486 PMC13082484

[35] Chai H , Sun C , Liu J , Sheng H , Zhao R , Feng Z . The relationship between ZEB1‐AS1 expression and the prognosis of patients with advanced gastric cancer receiving chemotherapy. Technol Cancer Res Treat. 2019;18:1533033819849069. 31072267 10.1177/1533033819849069 PMC6515840

[36] Zhou X , Fan YH , Wang Y , Wang F , Liu Y . Prognostic value of long non‐coding RNA ZEB1‐AS1 in Chinese cancer patients: A Meta‐analysis. Medicine. 2019;98:e15251. 31027073 10.1097/MD.0000000000015251 PMC6831238

[37] Wu Y , Ding M , Wei S , et al. The prognostic value of long noncoding RNA ZEB1‐AS1 on clinical outcomes in human cancer. J Cancer. 2018;9:3690‐3698. 30405838 10.7150/jca.27263 PMC6216015

[38] Hanahan D , Weinberg RA . Hallmarks of cancer: the next generation. Cell. 2011;144:646‐674. 21376230 10.1016/j.cell.2011.02.013

[39] Cheng R , Li N , Yang S , Liu L , Han S . Long non‐coding RNA ZEB1‐AS1 promotes cell invasion and epithelial to mesenchymal transition through inducing ZEB1 expression in cervical cancer. Onco Targets Ther. 2018;11:7245‐7253. 30425516 10.2147/OTT.S179937 PMC6203088

[40] Lv SY , Shan TD , Pan XT , et al. The lncRNA ZEB1‐AS1 sponges miR‐181a‐5p to promote colorectal cancer cell proliferation by regulating Wnt/beta‐catenin signaling. Cell Cycle. 2018;17:1245‐1254. 29886791 10.1080/15384101.2018.1471317 PMC6110576

[41] Li M , Guan H , Liu Y , Gan X . LncRNA ZEB1‐AS1 reduces liver cancer cell proliferation by targeting miR‐365a‐3p. Exp Ther Med. 2019;17:3539‐3547. 30988735 10.3892/etm.2019.7358 PMC6447761

